# Spruce Balm-Based Semisolid Vehicles for Wound Healing: Effect of Excipients on Rheological Properties and Ex Vivo Skin Permeation

**DOI:** 10.3390/pharmaceutics15061678

**Published:** 2023-06-08

**Authors:** Elisabeth Eichenauer, Martina Jozić, Sabine Glasl, Victoria Klang

**Affiliations:** 1 Division of Pharmacognosy, Department of Pharmaceutical Sciences, University of Vienna, Josef-Holaubek-Platz 2, 1090 Vienna, Austria; 2 Vienna Doctoral School of Pharmaceutical, Nutritional and Sport Sciences, University of Vienna, Josef-Holaubek-Platz 2, 1090 Vienna, Austria; 3 Division of Pharmaceutical Technology and Biopharmaceutics, Department of Pharmaceutical Sciences, University of Vienna, Josef-Holaubek-Platz 2, 1090 Vienna, Austria

**Keywords:** Norway spruce balm, *Picea abies*, rheology, semisolid, skin permeation, storage stability, wound healing ointment

## Abstract

The treatment of chronic wounds, an important issue with the growing elderly population, is increasingly hindered by antibiotic resistance. Alternative wound care approaches involve the use of traditional plant-derived remedies, such as purified spruce balm (PSB), with antimicrobial effects and the promotion of cell proliferation. However, spruce balm is difficult to formulate due to its stickiness and high viscosity; dermal products with satisfying technological properties and the scientific literature on this topic are scarce. Thus, the aim of the present work was to develop and rheologically characterize a range of PSB-based dermal formulations with different hydrophilic/lipophilic compositions. Mono- and biphasic semisolid formulations based on different compounds (petrolatum, paraffin oil, wool wax, castor oil, and water) were developed and characterized by their organoleptic and rheological measurements. A chromatographic method of analysis was established, and skin permeation data were collected for pivotal compounds. The results showed that the dynamic viscosity ranged from 10 to 70 Pas at 10/s for the different shear-thinning systems. The best formulation properties were observed for water-free wool wax/castor oil systems with 20% *w*/*w* PSB followed by different water-in-oil cream systems. Skin permeation through porcine skin was observed for different PSB compounds (e.g., pinoresinol, dehydroabietic acid, and 15-hydroxy-dehydroabietic acid) using Franz-type diffusion cells. The permeation potential of wool wax/castor oil- and lard-based formulations was shown for all the analyzed substance classes. The varying content of pivotal compounds in different PSB batches collected at different timepoints from different spruce individuals might have contributed to observed differences in vehicle performance.

## 1. Introduction

Wound healing is a complex mechanism involving many different cell types, growth factors, and cytokines. Hence, plenty of different factors may impair this wound healing process, especially chronic diseases, such as diabetes and obesity, but also acute infections and the process of aging. Approximately one billion people are affected by chronic wounds globally, with an increasing incidence due to the rising occurrence of antimicrobial resistance, metabolic syndrome, and a higher lifespan with accompanying comorbidities. Chronic wounds compromise patients’ quality of life and represent a monetary burden for healthcare systems worldwide [1,2]. Therefore, additional therapy options are urgently needed. Plants and extracts thereof have been used for centuries as traditional medicines and are indispensable in the treatment of various diseases and in the development of new drugs. They often contain several different compound classes, which can provide multitargeted effects. Especially in complex mechanisms such as wound healing, the presence of different substances with diverse activities, such as substances with antioxidant, anti-inflammatory, or antimicrobial properties, is highly beneficial [1].

**Figure 1:**
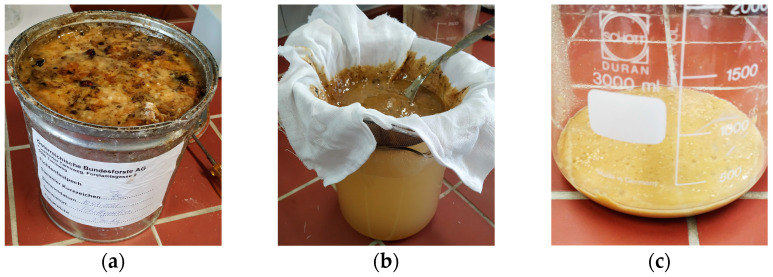
Purification process of Norway spruce balm: (**a**) Crude balm after collection from tree trunks, including impurities such as bark, needles, and insects. (**b**) The balm is placed in a linen cloth, which is then tied up and placed in boiling water. The balm becomes a viscous liquid and flows into the water; the impurities remain in the linen cloth. (**c**) After cooling down, the purified spruce balm (PSB) sinks to the bottom, and the water can be drained.

The Norway spruce (*Picea abies* (L.) H. Karst.) [3] produces, like other members of the family Pinaceae, resinous excretions for protection from pathogens such as bacteria and fungi [4]. The exudates can be divided into balms, the fresh, sticky, and kneadable excretions with a high content of essential oil, and resins, the brittle, nonvolatile excretions that do not contain essential oil and that harden after outflow [5]. The balms and resins of *Picea abies* have been used for centuries in the treatment of infected acute or chronic wounds and burns, but also onychomycosis. Traditionally, an ointment made from exudates processed with lard or butter was applied to the affected area [4]. In Austria, mostly the balm of the Norway spruce has had a long-standing tradition in folk medicine. After collecting the balm from tree trunks, impurities such as bark, needles, and insects can be detected (Figure 1a). Therefore, a purification process has to be conducted prior to further usage. For this purpose, the balm is placed in a linen cloth and is boiled in water (Figure 1b). The high temperature changes the balm’s consistency to a viscous liquid and permits it to pass through the linen tissue into the water, whereas the solid impurities remain in the cloth. After cooling down, the purified spruce balm (PSB) gathers at the bottom due to its higher density (Figure 1c), and the water can be removed through decantation [6,7,8,9]. After this purification step, the balm can be used to produce semisolid products such as ointments. Since 2016, the Austrian Pharmacopeia has contained a monograph entitled “Piceae abietis balsamum”. In 2019, the monograph “Unguentum balsami Piceae Abietis officinale”, an ointment consisting of 80 g of pig lard and 20 g of Norway spruce balm, was added [10].

The wound healing potential of Norway spruce balm has already been extensively investigated. A summary of the in vitro effects of PSB compounds that are relevant for wound healing applications is shown in Table 1. Wounds are often colonized by bacteria and fungi that impair the healing mechanism. The balm exhibits an antimicrobial effect against such microbes, such as methicillin-resistant *Staphylococcus aureus* and the dermatophyte *Trichophyton rubrum*. Two clinical trials with patients suffering from pressure ulcers or complicated surgical wounds successfully confirmed this wound healing potential in vivo [4,11,12,13,14,15]. In order to gain insight into the underlying mechanisms and substances responsible for this effect, the chemical composition of the balm has been investigated. The substance classes contained in Norway spruce balm are hydroxycinnamic acids, such as ferulic acid and caffeic acid; lignans, such as pinoresinol; and essential oil, which is mostly removed in the boiling step in the traditional method of preparation. The main compounds are different resin acids, which belong to the class of diterpene resin acids (DRAs). These acids can be hydroxylated or nonhydroxylated. Seven of these acids have the same molecular weight of 302 g/mol and only differ in the position of a double bond or in their stereochemistry [9,16,17,18]. Table 1 displays the most important substances identified in PSB and their bioactivities, which possibly contribute to the overall wound healing effect.

The different substance classes cover a large range of polarity. Diterpene resin acids, which form a major part of PSB, are apolar, whereas hydroxycinnamic acids and lignans possess more polar properties. Traditionally, the balm is processed with butter or lard, which results in very lipophilic formulations. The available PSB-containing ointment on the market follows this traditional preparation and is based on lard (Picea-Salbe^®^, Sico Pharma GmbH, Grafenwörth, Austria). Very little is known, however, about other options to produce semisolid vehicles, e.g., those with different polarities, optimized rheological properties, and storage stability. The potential to produce multiphase systems such as emulsions has not been explored yet, although different technologies would be a definite asset to targeting different wound healing applications. Moreover, different patient groups could be addressed with a larger portfolio of spruce-balm-based semisolid vehicles, as lard is not considered to be an ideal raw material in terms of standardized quality, chemical stability, or patient compliance.

Thus, the aim of this work was to develop and rheologically characterize a range of PSB-based dermal formulations with different hydrophilic/lipophilic compositions. Mono- and biphasic semisolid formulations based on different compounds (petrolatum, paraffin oil, wool wax, castor oil, and water) were developed and characterized by their organoleptic and rheological measurements. To our knowledge, the impact of PSB in semisolid vehicles on their rheological properties and storage stability has not been reported yet. Furthermore, permeation studies using an ex vivo porcine ear model [32,33] were conducted using the most promising formulation as well as the commercially available product Picea-Salbe^®^ (Sico Pharma GmbH, Grafenwörth, Austria). So far, there are no published data at all on the permeation potential of different PSB compounds. Therefore, the HPLC-DAD method was applied for the relative quantification of pivotal PSB compounds during diffusion cell studies to confirm and compare their permeation potential. The scope of this study was thus a first nonquantitative evaluation of the permeation of PSB compounds ex vivo; however, the exact quantification of skin permeation is planned to be performed in follow-up studies.

## 2. Materials and Methods

### 2.1. Materials

#### 2.1.1. Plant Material

Spruce balm from *Picea abies* (L.) H. Karst. was collected by the Österreichische Bundesforste AG (ÖBf, Austrian federal forests) in a forest in Lungau, Salzburg, Austria, in July 2021 and was provided by Sico Pharma GmbH, Grafenwörth, Austria. The batch was assigned the name PSB5 and was stored in a metal container at 4 °C.

Since the traditional method of preparation includes the step of boiling Norway spruce balm, 342 g of balm was sealed in a linen cloth and was placed in a beaker containing 2000 mL of deionized water heated up to approximately 90 °C. After boiling the balm for about one hour and letting the water cool down, the purified spruce balm (PSB) gathered at the bottom of the beaker, the water was removed through decantation, and the residue was dried and yielded 187 g of PSB.

#### 2.1.2. Other Materials

The commercial product Picea-Salbe^®^ (containing 20% *w*/*w* Norway spruce balm in lard, L200408) was kindly provided by Sico Pharma GmbH, Grafenwörth, Austria. Aerosil^®^ (KNC1914J14), paraffin oil PHQ (KNC0029J20), castor oil PHQ (KNC0504J21), petrolatum PHQ (KNC1132J19), and wool wax PHQ (KNC0113J20) were obtained from Herba Chemosan Apotheker AG, Vienna, Austria. Ultrabas^®^ (YY04P1H) was purchased from Bayer Austria GmbH, Vienna, Austria.

### 2.2. Preparation of Formulations

Different formulations were developed to incorporate 20% *w*/*w* of PSB. The respective compositions are given in Table 2. PSB was melted together with a lipophilic base in a water bath at 80 °C and was stirred afterward until the formulation was cool. If requested, distilled water was incorporated stepwise in small portions. For the preparation of an oleogel with colloidal silica (Aerosil^®^), disperse powder was incorporated stepwise in a PSB/castor oil mixture in small portions. Using Ultrabas^®^ as ointment base, PSB was melted with castor oil, and the resulting cool homogenate was mixed stepwise with Ultrabas^®^. All ointments were produced in triplicate and were stored in gel tubes under light protection. Each formulation was divided in half to give two batches, which were stored either at room temperature (23 °C) or in the refrigerator (8 °C) to assess the effect of different storage temperatures.

### 2.3. Rheological Characterization and Stability

The rheological properties of the produced semisolid ointments were analyzed with a modular compact rheometer (MCR 302 with viscotherm VT 2 thermostatic control system, Anton Paar GmbH, Graz, Austria). Data were analyzed using the RheoCompass Professional^®^ 1.26 software (Anton Paar GmbH, Graz, Austria). Flow curves were recorded at room temperature of 23 ± 0.2 °C with a cone–plate measuring system (CP-25, diameter of 25 mm, angle of 2°) using ~1.0 g of the respective vehicle. Dynamic viscosity η was determined based on dependence of shear rate at 1 to 100 s^−1^. For stability monitoring at different storage temperatures, respectively, three samples of each formulation type were filled in gel tubes after production. For initial assessment, all ointments were stored under light protection at 23 °C for 24 h to allow for equilibrium and were then characterized. Subsequently, the samples were either stored at 23 °C (room temperature, *n* = 3 per type) or 8 °C (refrigerated storage, *n* = 3 per type) and were analyzed in regular intervals using the same parameters over six months. Cooled samples were allowed to acclimatize for six hours at 23 °C before each measurement.

### 2.4. Skin Permeation

#### 2.4.1. Diffusion Cell Setup

Skin permeation studies were performed using dermatomed porcine ear skin and static Franz-type diffusion cells (PermeGear, Hellertown, PA, USA, permeation area of 0.95 cm^2^, acceptor volume of 2 mL). Porcine ears were obtained after sacrifice from a local abattoir (Johann Gantner GmbH, Gemeinde Hollabrunn, Austria, male and female animals at six months of age). The ears were cleaned under running water and were dried and stored at −18 °C until use.

Fresh vehicles were prepared 48 h before the start of the experiments. Skin from the dorsal side of the ears was cut to a thickness of 400 µm with a dermatome (Aesculap GA 630 DBP, Aesculap AG, Tuttlingen, Germany). Hair was removed with scissors. The skin was cut into 1 × 1 cm pieces and was clamped between donor and acceptor chamber. The acceptor medium consisted of a mixture of phosphate-buffered saline and ethanol (60:40% *v*/*v*) to ensure sink conditions. The phosphate-buffered saline was prepared as described in the Pharmacopoeia Europaea (0.012 M, pH of 7.4, 0.238% *w*/*w* sodium monohydrogen phosphate, 0.019% *w*/*w* potassium dihydrogen phosphate, and 0.8% *w*/*w* sodium chloride). Before starting the experiments, the transepidermal water loss (TEWL) was measured with a closed-chamber device and a coupling adapter (Aquaflux^®^ AF200, Biox Systems Ltd., London, UK) to ensure consistent skin quality and barrier function.

#### 2.4.2. Skin Permeation of Bioactive Substances

The ointments were applied to the donor chambers at infinite dose conditions (500 mg of formulation). The donor compartment and sampling arm were occluded with Parafilm to prevent evaporation of sample or acceptor fluid. The diffusion cells were kept in a water bath at 32 °C to simulate skin surface temperature. Magnetic stirring was employed to avoid concentration layers within the acceptor fluid. Cells were regularly checked for air bubbles. After predefined intervals (2, 4, 6, 8, 24, 28, and 32 h), an aliquot of 200 μL of acceptor medium was taken for analysis and was replaced with fresh acceptor medium at 32 °C.

The most suitable formulation was chosen for analysis of permeation potential as well as the commercial product Picea-Salbe^®^. Three individual permeation experiments with *n* = 3 cells were performed for formulations and controls. The final results depict mean values ± SDs of *n* = 9 cells per formulation. As controls, the respective ointment bases without PSB as well as the plain acceptor medium alone were used.

### 2.5. High-Performance Liquid Chromatography

For the analysis of samples drawn from Franz diffusion cells, a Shimadzu HPLC instrumentation with diode array detection (SHIMADZU Autosampler SIL-20AC HAT, SHIMADZU Column Oven CTO-20AC, SHIMADZU Communications Bus Module CBM-20A, SHIMADZU Degasser DGU-20A5, SHIMADZU Diode Array Detector SPD-M20A, SHIMADZU Liquid Chromatograph LC-20AD, SHIMADZU LabSolutions Software 5.81, Shimadzu, Kyoto, Japan) was used. A reversed-phase C18 column (LiChrospher 100 RP18e, 5 μm, 250 × 4 mm, Merck KGaA, Darmstadt, Germany) was employed as stationary phase. Double-distilled water + 0.1% formic acid (solvent A) and acetonitrile + 0.1% formic acid (solvent B) served as mobile phase, and gradient elution at 1 mL/min flow rate was applied as follows: 15% solvent B rose to 95% solvent B within 45 min (rate of 1.77%/min) followed by 5 min washing step at 98% solvent B and re-equilibration for 10 min with 15% solvent B. Samples drawn from Franz diffusion cells were centrifuged, and 10 μL were injected into the system. PSB samples were dissolved in acetone to a final concentration of 5 mg/mL, and 2 μL were analyzed. Since the different substance classes exhibit different absorption maxima but can be seen at 190 nm, all chromatograms were analyzed at 190 nm. Peak areas (AUC) were obtained using the SHIMADZU LabSolutions Software 5.81 (Shimadzu, Kyoto, Japan) and were further utilized for interpretation of data.

### 2.6. Statistics

Results are expressed as statistical means with standard deviations. Data analysis was performed using GraphPad Prism 9.4.1 software (GraphPad Software, San Diego, CA, USA). Parametric data were analyzed using Student’s *t*-test or ANOVA and Tukey’s post hoc test. Nonparametric data were analyzed using the Mann–Whitney test or Kruskal–Wallis test with Dunn’s multiple comparison test as post hoc test. Statistical significance was expressed by minimum of *p* < 0.05 (*), *p* < 0.01 (**), and *p* < 0.001 (***).

## 3. Results

### 3.1. Preparation and Organoleptic Appearance of Formulations

**Figure 2:**
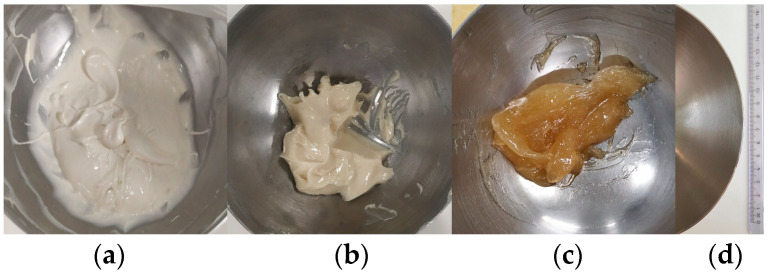
Optical appearance of different formulations: (**a**) wool wax/castor oil; (**b**) Ultrabas/castor oil; (**c**) Aerosil/castor oil. All formulations were prepared using a smooth mortar ((**d**) upper diameter of 12 cm) and pestle.

By using different ointment bases, 20% *w*/*w* PSB-containing ointments with varying hydrophilic/lipophilic compositions were prepared. The incorporation of PSB into petrolatum and paraffin oil led to the caking of the balm, suggesting the need for further additives in hydrocarbon-based PSB ointments. This formulation was excluded from further experiments. Preparation with wool wax and castor oil/water, Ultrabas^®^ and castor oil, and colloidal silica (Aerosil^®^) and castor oil resulted in dermal formulations with homogenous optical properties and a whiteish to brownish appearance (Figure 2).

### 3.2. Rheological Properties and Stability

**Figure 3:**
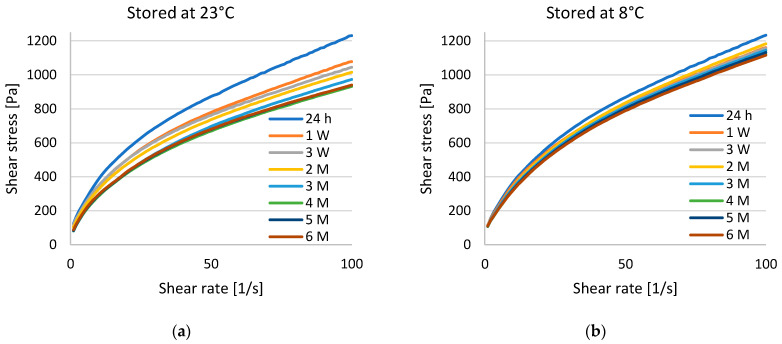
Flow curves of (**a**) wool wax/castor oil stored at room temperature (23 °C) and (**b**) wool wax/castor oil stored in the refrigerator (8 °C). The colored curves are means of *n* = 3 samples analyzed at room temperature (23 °C) at a shear rate of 1–100 s^−1^. SDs are omitted for better visualization. The refrigerated samples were left to acclimate for six hours at 23 °C before each measurement. Abbreviations: h = hour/s, W = week/s, and M = month/s.

Flow curves were established for all the developed vehicles by plotting the shear stress τ against the shear rate γ. The flow curves of the preparation of wool wax/castor oil are depicted in Figure 3; the flow curves of wool wax/water and Ultrabas/castor oil can be found in Supplementary Figures S1 and S2. The formulation of Aerosil/castor oil was excluded for methodological reasons after its initial assessment (it had a paste-like consistency due to its high powder content; the product was deemed unsuitable for use on open wounds).

**Figure 4:**
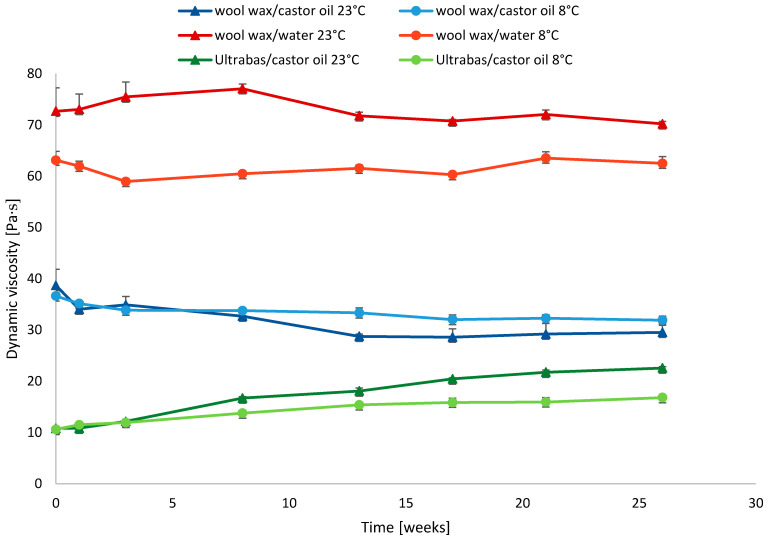
Effect of storage time and temperature on dynamic viscosity (η, in Pas) of semisolid vehicles with 20% *w*/*w* PSB at a shear rate of 10 s^−1^. Values are means ± SDs of *n* = 3 samples.

Furthermore, viscosity curves were established by plotting the dynamic viscosity η against the shear rate γ. The dynamic viscosity in Pas at a shear rate of 10 s^−1^ was monitored during storage at two different conditions (room temperature of 23 °C and refrigerated temperature of 8 °C) and served to compare the rheological storage stability of the different formulations (Figure 4).

The initial dynamic viscosity was calculated for all the samples of the three formulations independent from the temperature, and it was highest for the wool wax/water formulation followed by wool wax/castor oil and finally Ultrabas/castor oil (68.05 ± 5.88 > 37.64 ± 2.38 > 10.63 ± 0.35 Pas at 10 s^−1^, *p* < 0.0001, *n* = 6).

The formulation of wool wax/castor oil visually exhibited the most stable formulation properties and storage stability. After an initial structural stabilization period over the first week, the dynamic viscosity only showed a slow decrease over a storage period of six months (Figure 4). The flow curves were highly reproducible for wool wax/castor oil (Figure 3); no visual signs of the structural rearrangement or physical separation of PSB from the vehicle were observed. As can be derived from the flow curves (Figure 3), the dynamic viscosity decreased with increasing shear rates. This corresponds to pseudoplastic, i.e., shear-thinning, flow behavior. After six months, the dynamic viscosity of the refrigerated product generally remained at a higher value than that of the product that was stored at room temperature (Figure 4). At 23 °C, the dynamic viscosity at 10 s^−1^ changed from the initial 36.68 ± 3.15 Pas to 29.52 ± 1.63 Pas after six months (*p* < 0.05). At 8 °C, the values changed from the initial 36.60 ± 0.67 Pas to 31.90 ± 0.78 Pas after six months (*p* < 0.01). This, however, was owed to the reorganization phase within week 1 after which only minor changes were observed, especially with respect to cooled storage.

The multiphase system based on wool wax/water showed the first signs of visual phase separation within the first weeks of storage. The flow curves generally showed pseudoplastic behavior, but they showed less consistent shear thinning during measurement (Supplementary Figure S1) than the wool wax/castor oil system. A thickening effect during storage was visible due to a loss of water, which was more pronounced for the formulations stored at room temperature. Although the measured values of the dynamic viscosity at 10 s^−1^ remained largely unaffected after six months for both the formulations stored at 23 °C (72.68 ± 4.57 vs. 71.19 ± 1.99 Pas, *p* > 0.05) and at 8 °C (63.43 ± 1.58 vs. 62.49 ± 1.38 Pas, *p* > 0.05), the homogeneity of the formulations was obviously impaired, and the reproducibility between the batches was limited under lab-scale compounding conditions. The observed separation of water was a disruptive factor for the rheological measurements, and it could be deduced from the fluctuating values of the dynamic viscosity at 10 s^−1^ during rheological monitoring during storage (Figure 4).

For the Ultrabas/castor oil formulation, a clear tendency towards an increasing dynamic viscosity was observed during storage (Figure 4). As for the wool wax/water system, the flow curves indicated pseudoplastic flow behavior, but with strong irregularities during measurement (Supplementary Figure S2). Since the water content of Ultrabas^®^ is lower than that of the wool wax/water-based product, more homogeneous formulation properties were visually observed. Nonetheless, a slow separation of water from the main structure and internal structural changes could be deduced from rheological monitoring, leading to an apparent increased dynamic viscosity. This effect was more pronounced for the formulation stored at room temperature after six months. The dynamic viscosity at 10 s^−1^ at 23 °C increased from 10.78 ± 0.38 to 23.15 ± 1.22 Pas (*p* < 0.001). At 8 °C, the increase still amounted to a change from 10.48 ± 0.28 to 16.38 ± 0.81 Pas (*p* < 0.01).

In summary, the wool wax/castor oil-based formulation exhibited the most homogeneous formulation properties and the highest rheological stability. Thus, this vehicle was selected for evaluation against a marketed product in skin diffusion studies.

### 3.3. Solubility and Choice of Acceptor Medium

**Figure 5:**
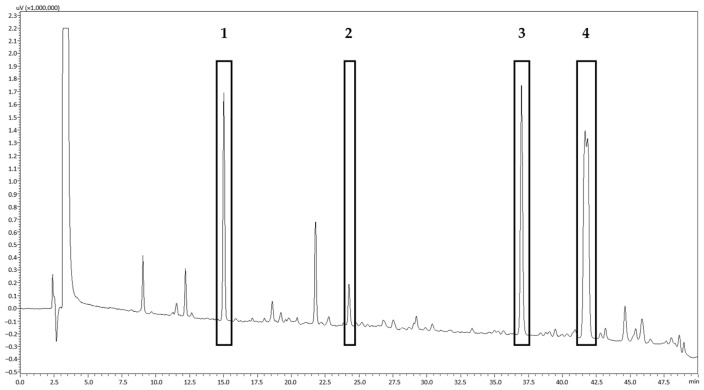
HPLC-DAD chromatogram of PSB (batch PSB5) at 190 nm (balm dissolved in acetone, final concentration of 5 mg/mL, 2 μL were injected); stationary phase was RP18e, and mobile phase was water + 0.1% formic acid and acetonitrile + 0.1% formic acid. The peaks of substances, which were chosen for analysis of their permeation potential, were assigned through co-chromatography with pure reference substances. Peak 1 is pinoresinol, Peak 2 is 15-hydroxy-dehydroabietic acid, and Peak 3 is dehydroabietic acid. Peak 4 contains 7 different DRAs, which all share the same molecular weight of 302 g/mol and cannot be separated using this method due to their structural similarities.

Diffusion cell studies were conducted to probe for the permeation potential of important PSB compounds through intact porcine ear skin in an ex vivo setup. Focus was placed on four important groups of PSB compounds: pinoresinol (**1**), 15-hydroxy-dehydroabietic acid (**2**), dehydroabietic acid (**3**), and DRAs as summarized by the content of seven diterpene resin acids with a molecular weight of 302 g/mol (**4**) (Figure 5).

Since the majority of these compounds is apolar in nature and since their solubility in an aqueous phosphate buffer as the standard acceptor medium is thus limited, two different solvents were tested: 50 mg of PSB were dissolved in each acceptor medium (either ethanol or propylene glycol in phosphate-buffered saline (40 + 60)), was shaken for 24 h at 30 °C, and was centrifuged afterwards, and 5 µL were analyzed through HPLC-DAD. The obtained AUCs can be found in Supplementary Figure S3. Since a larger amount of substances, especially the more polar pinoresinol and 15-hydroxy-dehydroabietic acid, was detected in the acceptor medium containing ethanol, the solubility of the substances was higher in ethanol than in propylene glycol, and, therefore, ethanol + phosphate-buffer saline (40 + 60) was used as the acceptor medium.

Furthermore, permeation experiments with the same setup as those with the PSB-containing ointments were conducted using no ointment or only the ointment base (lard or wool wax/castor oil) in order to identify possible overlapping peaks in the respective HPLC chromatograms deriving from variables other than PSB itself. Since no additional peaks other than the expected peaks were detected, it was assumed that the detected mass permeation of these compounds was not adulterated by other factors (Supplementary Figure S4).

### 3.4. Skin Permeation of PSB Compounds

**Figure 6:**
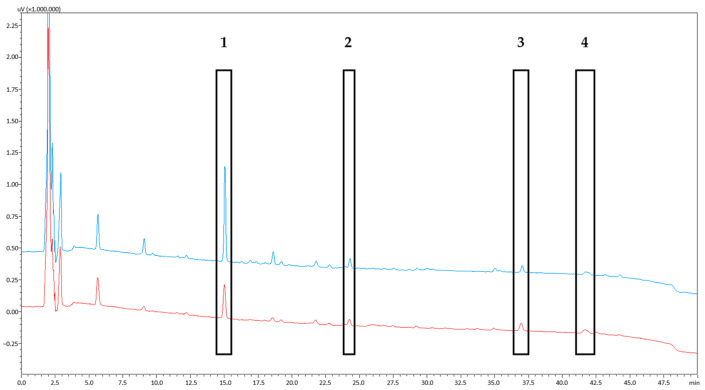
HPLC-DAD chromatograms (190 nm) of samples drawn after 32 h of skin permeation experiments of 20% PSB in lard (blue) and 20% PSB in wool wax/castor oil (red). The assigned peaks mark the substances, which were analyzed in depth (pinoresinol (**1**), 15-hydroxy-dehydroabietic acid (**2**), dehydroabietic acid (**3**), and 302 g/mol DRAs (**4**)).

**Figure 7:**
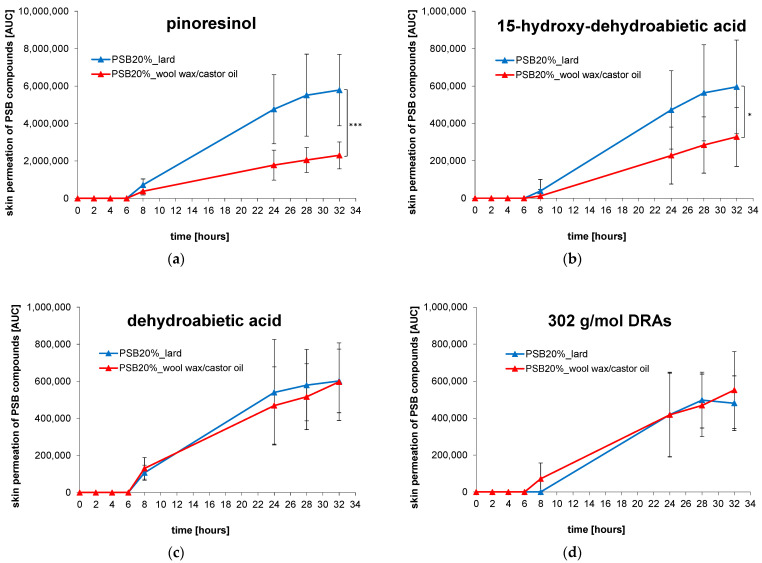
Skin permeation of PSB compounds (pinoresinol (**a**), 15-hydroxy-dehydroabietic acid (**b**), dehydroabietic acid (**c**), and 302 g/mol DRAs (**d**)) from different ointments. Data represent means of *n* = 9 cells ± SDs. Statistically significant differences between formulations are marked for AUC values after 32 h with * for *p* < 0.05, and *** for *p* < 0.001.

Skin permeation experiments were conducted through the application of the wool wax/castor oil-based formulation and the lard-based Picea-Salbe^®^. The PSB compounds were analyzed over 32 h of experiment time ex vivo. The relative quantification of mass permeation was possible based on the obtained AUC values (Figure 6, measured at the timepoint of 32 h). The cumulative permeated amounts of pinoresinol, 15-hydroxy-dehydroabietic acid, dehydroabietic acid, and the DRAs were established using the obtained AUCs and were plotted against the experiment time for the comparison of the two tested products (Figure 7). For pinoresinol (Figure 7a), significantly higher total permeated amounts were observed from the lard ointment than from the wool wax/castor oil ointment (*p* < 0.001). For 15-hydroxy-dehydroabietic acid (Figure 7b), a similar trend was observed despite large standard deviations in the data set (still, *p* was < 0.05). For dehydroabietic acid and the DRAs (Figure 7c,d), the total permeated amounts were highly comparable from the two different ointments (*p* > 0.05 in both cases).

In addition, the steady-state flux in AUC/cm^2^/h was calculated through linear regression for the individual cells after the respective lag time (6 or 8 h). The mean relative mass flux of the different PSB compounds was calculated through linear regression to compare the two vehicles (Table 3); since no absolute quantification was yet possible, the mass flux is termed “relative” in this context. The results confirmed a significantly higher mass flux for pinoresinol and 15-hydroxy-dehydroabietic acid from the lard-based ointment and a highly similar mass flux for dehydroabietic acid and the DRAs from the two ointments.

**Figure 8:**
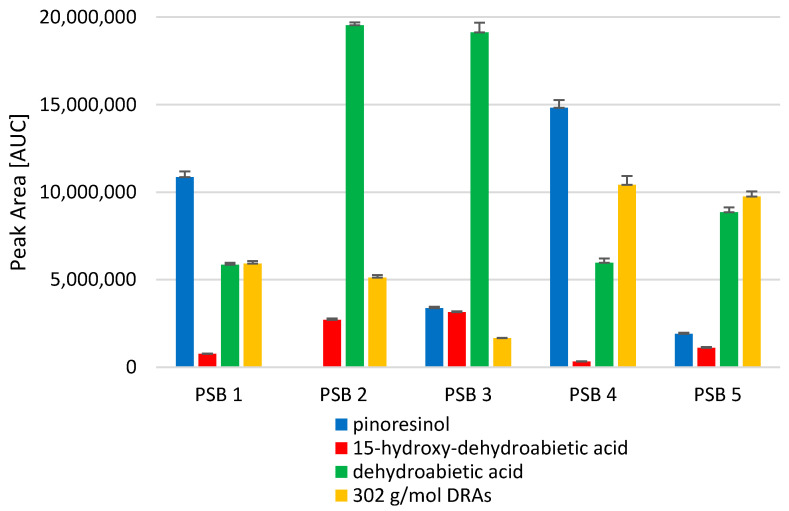
Relative content (AUC) ± SDs of substances in five different PSB batches (means of *n* = 3 injections); batches were collected in different areas and at different timepoints around Austria.

When comparing the two products, it has to be kept in mind that different plant materials were used for their preparation. For the wool wax/castor oil-based formulation, PSB5 with a known composition was used (Figure 5). The commercially available product Picea-Salbe^®^, however, consists of a Norway spruce balm with an unknown composition. The detected differences in permeation between the two products may, on the one hand, result from the different solubilities of the compounds in their respective underlying ointment base. On the other hand, they may be due to the varying contents of the substances in the different spruce balm samples. In order to show the heterogeneity of Norway spruce balms, five different batches were analyzed via HPLC-DAD (Figure 8). These batches were collected at different timepoints and in different areas around Austria (Supplementary Table S1, PSB5 was used for the permeation experiments in wool wax/castor oil), and they demonstrate the highly varying relative contents of the substances of interest.

## 4. Discussion

### 4.1. Preparation of PSB Formulations and Storage Stability

The successful incorporation of PSB into semisolid dermal vehicles is challenging due to the large diversity of phytochemicals present in the balm and the peculiar rheological behavior of the balm itself after harvesting. Both the monograph in the Austrian Pharmacopeia dealing with PSB ointments as well as the commercial product Picea-Salbe^®^ rely on a very traditional production method: the purified spruce balm is either mixed with lard or butter after heating, and stirred until a homogeneous mixture at room temperature is obtained. However, these animal fats are not commonly available in pharmaceutical quality and/or are not sufficiently standardized, thus not meeting today’s technological standards. As a result, the physicochemical formulation parameters of the produced semisolid vehicles as well as patient compliance may vary. To overcome these issues, alternative ointment bases should be explored for the delivery of PSB in wound healing applications.

Interestingly, however, lard itself has been shown to exhibit anti-inflammatory properties [34] and might, therefore, contribute to an overall wound healing effect. An in vivo study on the healing of castration wounds in piglets compared lard alone to a PSB-/lard-based ointment. In this study, the use of a pure ointment base did not lead to the reduction of proinflammatory factors or faster wound healing [35]. Thus, other ointment bases should be considered as a replacement.

In Finland, a medical product containing the resin of *Picea abies* is available, which is based on petrolatum, paraffin oil, and further additives for stabilization. A simple prototype formulation was developed at the beginning of this study using this formulation approach but was unsuccessful and was thus excluded. In further studies, a more complex formulation based on hydrocarbon-based products will be explored as an alternative to lard.

Wool wax was chosen as an ointment base due to its lipophilic texture and high emulsifying properties. Castor oil was added for a smoother texture and easier manual preparation. The rheological analysis of the preparation of wool wax/castor oil showed that the formulation required approximately one week to reach its quiescent structure. Over six months, the refrigerated product showed a higher rheological stability than the product stored at room temperature. As the most promising vehicle, the wool wax/castor oil ointment was selected as a candidate for the first diffusion cell experiments using porcine ear skin to evaluate the skin permeation potential of the PSB compounds ex vivo.

Wool wax was combined with water instead of castor oil in order to create a more hydrophilic vehicle, a semisolid oil-in-water cream stabilized by the nonionic wool wax surfactants. It should be noted that the presence of 20% *w*/*w* water might lead to increased microbial contamination during storage. Spruce balm incorporated in an apolar vehicle is considered to possess sufficient antimicrobial activity for storage over six months, as indicated by the Austrian Pharmacopoeia. However, this should be investigated in future studies for multiphase vehicles to ensure the safety of the products.

Ultrabas^®^ is a manufactured ointment base that is popular in the pharmaceutical compounding of dermatological products in Austria. From a technological viewpoint, it is a water-in-oil emulsion based on hydrocarbons, distilled water, and nonionic surfactants. Since it should not be heated during its formulation processes, castor oil was used to facilitate the manual incorporation of PSB in this study.

Both of the multiphase vehicles containing an additional water phase, the wool wax/water system and the Ultrabas/castor oil system, showed decreased rheological stability due to a slow separation of water from the main ointment structure. This effect was more pronounced for creams based on wool wax/water, where a fluctuating dynamic viscosity was observed during storage. For Ultrabas/castor oil systems, a steady increase in the dynamic viscosity showed a slower but consistent separation of water from the main cream structure during storage, leading to the thickening of the remaining cream system.

It is known that water-in-oil emulsions show technological incompatibilities with phenolic entities. The presence of phenolic compounds leads to a concentration-dependent destabilization of the emulsion system through H-bonding with nonionic surfactants. As a result, the separation of water, coagulation, and phase separation may occur. Several substances found in Norway spruce balm, such as pinoresinol or several hydroxycinnamic acids, exhibit phenolic structures and might lead to this undesired physicochemical interaction in the cream formulations. Consequently, storage stability is decreased.

Finally, an oleogel preparation based on colloidal silica (Aerosil^®^) was developed. The incorporation of PSB at 20% *w*/*w* into the gel led to a homogenous product. Colloidal silica should not be used on open wounds, but it might be an alternative in the treatment of dry skin or fissures. This approach will be taken up in separate studies since a more thorough rheological examination is required for the highly viscous product.

### 4.2. Permeation of Bioactive Substances

Two ointments (wool wax/castor oil and Picea-Salbe^®^) were investigated concerning the permeation potential of the bioactive substances (pinoresinol, 15-hydroxy-dehydroabietic acid, dehydroabietic acid, and the 302 g/mol DRAs). First, the samples drawn from diffusion cells containing only the respective ointment base or no ointment at all were analyzed in order to prove the absence of detected peaks with the same retention time as the wanted PSB components.

Pinoresinol and dehydroabietic acid were already detectable in all the samples after 8 h, whereas 15-hydroxy-dehydroabietic acid and the 302 g/mol DRAs could often only be detected after 24 h. This might be due to the original content in the ointment. The analysis of the different PSB samples showed that the relative content of the respective substances varied in the different batches. Not only the area of collection, but also the date of collection seemed to have an impact on the content of the compounds. This fact underlines the importance of different analytical methods for quality control [16]. Since it is not known which PSB was used for the medical device Picea-Salbe^®^, the differences in the permeation studies may be due to the variety in the content found in the different balms.

On the other hand, the differences may be due to the varying polarities of the substances (pinoresinol XLogP3-AA: 2.3; 15-hydroxy-dehydroabietic acid XLogP3-AA: 4.2; dehydroabietic acid XLogP3-AA: 5.6; sandaracopimaric acid as the representative of the 302 g/mol DRAs XLogP3-AA: 5.5) and, therefore, their solubility in the two different vehicles (the ointment basis and acceptor medium). Pinoresinol and 15-hydroxy-dehydroabietic acid exhibit smaller logP values than the DRAs and are therefore more likely to permeate from a lipophilic ointment into a more hydrophilic acceptor medium. The next step will be the absolute quantification of the components in crude PSB as well as in the acceptor medium after permeation experiments using an internal standard.

In a follow-up study it will be clarified whether the two different vehicles are responsible for the differences found in the release of the substances using the same batch of PSB, or whether they approximate and the varying content in the balm itself is responsible for these differences. In the latter case, it has to be excluded that lard is crucial for the wound healing effect of the traditional PSB ointment. If so, wool wax/castor oil represents a more standardized, stable, and animal-friendly alternative to lard in the monography of the Austrian Pharmacopeia as well as in the preparation of medical devices.

### 4.3. Implications for the Successful Formulation of Semisolid Products with Plant-Based Materials

The present study shows that it is of the utmost importance for an informed formulation design of plant-based products to consider the presence of phenolic compounds as well as charged compounds and surface-active substances, especially in multiphase systems. Technological incompatibilities due to ionic interactions or H-bonding are dependent on the total concentration of individual plant compounds and may not lead to the immediate phase separation of emulsion systems. However, masked incompatibilities may ultimately promote physical destabilization and thus impair storage stability as well.

## 5. Conclusions

A wool wax/castor oil formulation was found to be a suitable alternative to traditional lard as a semisolid base for the production of Norway spruce balm ointments (20% *w*/*w* PSB). Visual and rheological monitoring confirmed a satisfying physical stability over six months. Refrigerated storage at 8 °C led to the best results, especially in the case of the other tested cream formulations. Ex vivo experiments showed the permeation potential of the bioactive PSB compounds from the newly developed semisolid formulation and the commercial reference product Picea-Salbe^®^. As the next step, the impact of the vehicle composition will be investigated using the same PSB batch, and the absolute quantification of the permeated substances will be targeted.

## Figures and Tables

**Table 1:** Main compounds of purified spruce balm (PSB), respective substance classes, and biological effects that may contribute to the overall wound healing properties.

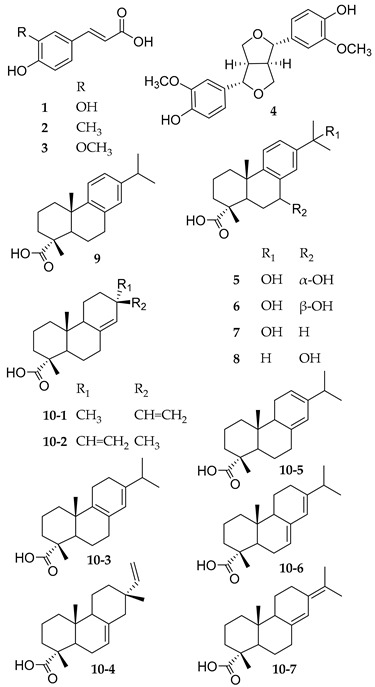				
		**Substance**	**Structure class**	**Effects contributing to wound healing**
	**1**	Caffeic acid	Hydroxy- cinnamic acids	antioxidant, antimicrobial, and anti-inflammatory effects [19]
	**2**	p-Coumaric acid		
	**3**	Ferulic acid		
	**4**	Pinoresinol	Lignans	antifungal [20], antioxidant, anti-inflammatory [21], and re-epithelialization boosting effects [9]
	**5**	7α,15-Dihydroxy-	Hydroxylated resin acids	re-epithelialization boosting [9], antimicrobial [22], and anti-inflammatory effects [23]
		dehydroabietic acid		
	**6**	7β,15-Dihydroxy-		
		dehydroabietic acid		
	**7**	15-Hydroxy- dehydroabietic acid		
	**8**	7-Hydroxy- dehydroabietic acid		
	**9**	Dehydroabietic acid	Diterpene resin acids (300 Da)	re-epithelialization boosting [9], anti-inflammatory [23], and antimicrobial effects [24]
	**10-1**	Pimaric acid	Diterpene resin acids (302 Da)	re-epithelialization boosting [9], antimicrobial [24,25,26,27], anti-inflammatory [28,29,30], and antioxidant effects [31]
	**10-2**	Sandaracopimaric acid		
	**10-3**	Palustric acid		
	**10-4**	Isopimaric acid		
	**10-5**	Levopimaric acid		
	**10-6**	Abietic acid		
	**10-7**	Neoabietic acid		
			

**Table 2:** Composition in % *w*/*w* of the prepared ointments with 20% *w*/*w* PSB. All formulations were produced in triplicate (*n* = 3).

Formulation	Composition	% *w*/*w*
petrolatum/paraffin	petrolatum	60
	paraffin oil	20
	PSB	20
wool wax/castor oil	wool wax	60
	castor oil	20
	PSB	20
wool wax/water	wool wax	60
	distilled water	20
	PSB	20
Ultrabas/castor oil	Ultrabas^®^	73.33
	castor oil	6.66
	PSB	20
Aerosil/castor oil	Aerosil^®^	7
	castor oil	73
	PSB	20

**Table 3:** Mean relative mass flux in AUC/cm^2^/h ± SDs of the different PSB compounds (** *p* < 0.01, *** *p* < 0.001).

PSB Compound	Vehicle	Mean Flux Based on AUC/cm^2^/h	Enhancement Factor	Sign.	Mean R^2^
pinoresinol	wool wax/castor oil	87.258 ± 27.553	2.8	***	0.973 ± 0.030
	lard	243.842 ± 93.731			0.989 ± 0.015
15-hydroxy-dehydroabietic acid	wool wax/castor oil	13.606 ± 6.196	1.8	**	0.985 ± 0.014
	lard	25.558 ± 9.673			0.988 ± 0.010
dehydroabietic acid	wool wax/castor oil	21.848 ± 7.791	1.1	n.s.	0.964 ± 0.026
	lard	24.041 ± 7.099			0.985 ± 0.012
302 g/mol DRAs	wool wax/castor oil	21.797 ± 7.567	1.1	n.s.	0.985 ± 0.023
	lard	22.847 ± 7.299			0.960 ± 0.035

## Data Availability

Not applicable.

## Supplementary Materials

The following supporting information can be downloaded at https://www.mdpi.com/article/10.3390/pharmaceutics15061678/s1: Figure S1: Flow curves of (a) wool wax/water stored at room temperature (23°C) and (b) wool wax/water stored in the refrigerator (8 °C). The colored curves are means of *n* = 3 formulations analyzed at room temperature (23 °C) at a shear rate of 1–100 s^−1^. SDs are omitted for better visualization. The refrigerated samples were left to acclimate for six hours at 23 °C before each measurement. Abbreviations: h = hour/s, W = week/s, and M = month/s. Figure S2: Flow curves of (a) Ultrabas/castor oil stored at room temperature (23 °C) and (b) Ultrabas/castor oil stored in the refrigerator (8 °C). The colored curves are means of *n* = 3 formulations analyzed at room temperature (23 °C) at a shear rate of 1–100 s^−1^. SDs are omitted for better visualization. The refrigerated samples were left to acclimate for six hours at 23 °C before each measurement. Abbreviations: h = hour/s, W = week/s, and M = month/s. Figure S3: Comparison of the relative solubility of PSB compounds in different acceptor media. AUCs were obtained after dissolving an identical surplus amount of purified spruce balm (PSB) in either phosphate-buffered saline/ethanol at 60:40% *v*/*v* (blue bars) or phosphate-buffered saline/propylene glycol at 60:40% *v*/*v* (red bars). Figure S4: HPLC-DAD chromatograms of samples drawn from permeation experiments after 32 h using no ointment (red), lard without PSB (green), and wool wax/castor oil without PSB (blue) at 190 nm. Table S1: Batch numbers (PSB), internal assignment (FP), date of harvest, and harvest location of analyzed PSB batches.
