# Supplementary material for: Spruce Balm-Based Semisolid Vehicles for Wound Healing: Effect of Excipients on Rheological Properties and Ex Vivo Skin Permeation
Source: Pharmaceutics. 2023 Jun 8;15(6):1678. doi: 10.3390/pharmaceutics15061678 (PMC10302299; doi:10.3390/pharmaceutics15061678)
Supplement: Supplementary file 1 [file pharmaceutics-15-01678-s001.zip › pharmaceutics-2378154-supplementary.pdf]

Supplementary Materials:

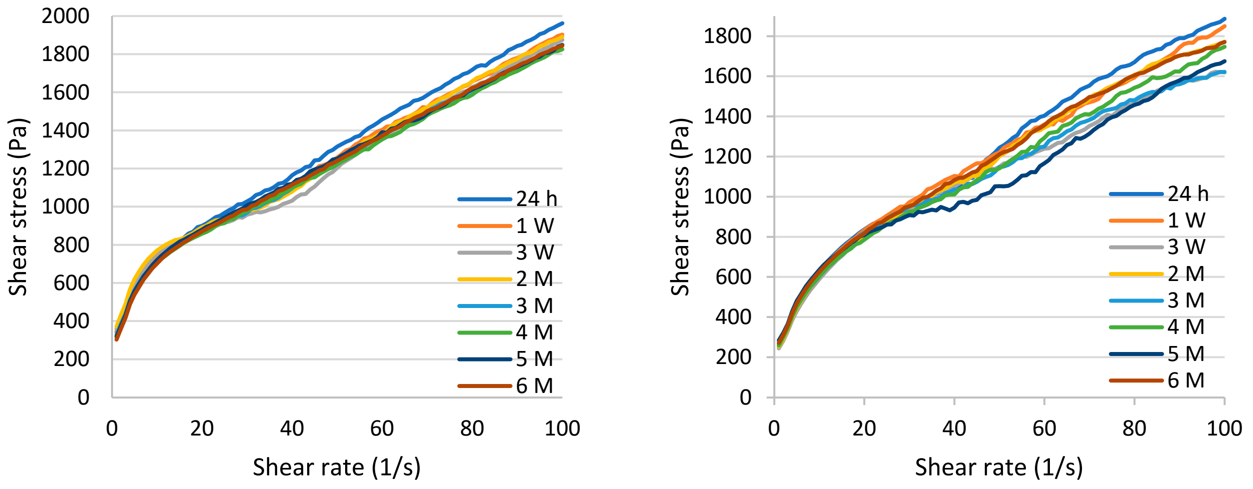

(**a**) (**b**)

Figure S1. Flow curves of (**a**) wool wax/water stored at room temperature (23°C) and (**b**) wool wax/water stored in the refrigerator (8°C). The colored curves are means of n = 3 formulations analyzed at room temperature (23°C) at a shear rate of 1-100 s^{-1}. SDs are omitted for better visualization. The refrigerated samples were left to acclimate for six hours at 23°C before each measurement. Abbreviations: h = hour/s, W = week/s, and M = month/s.

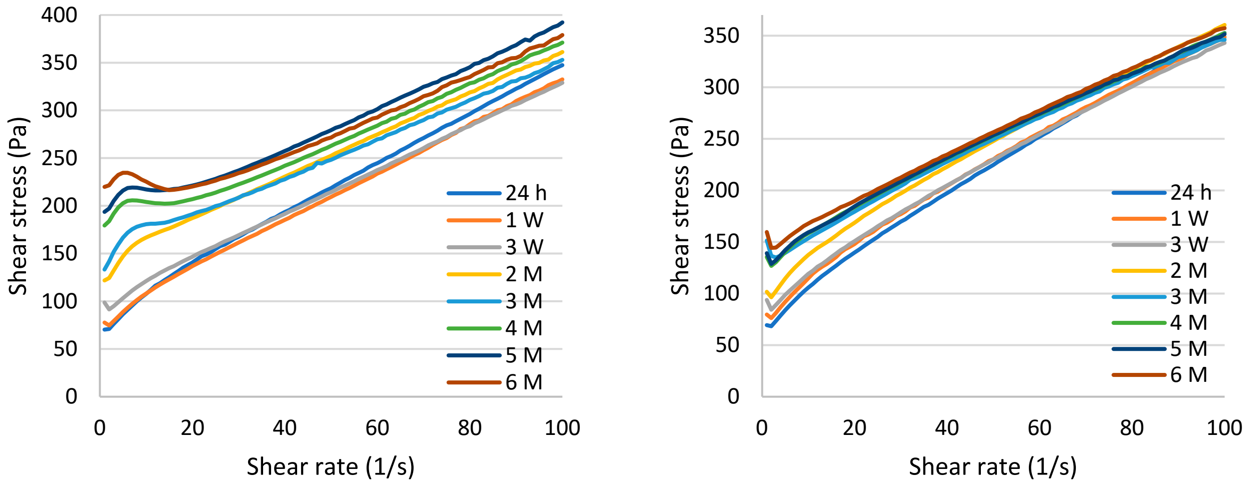

(**a**) (**b**)

Figure S2. Flow curves of (**a**) Ultrabas/castor oil stored at room temperature (23°C) and (**b**) Ultrabas/castor oil stored in the refrigerator (8°C). The colored curves are means of n = 3 formulations analyzed at room temperature (23°C) at a shear rate of 1-100 s^{-1}. SDs are omitted for better visualization. The refrigerated samples were left to acclimate for six hours at 23°C before each measurement. Abbreviations: h = hour/s, W = week/s, and M = month/s.

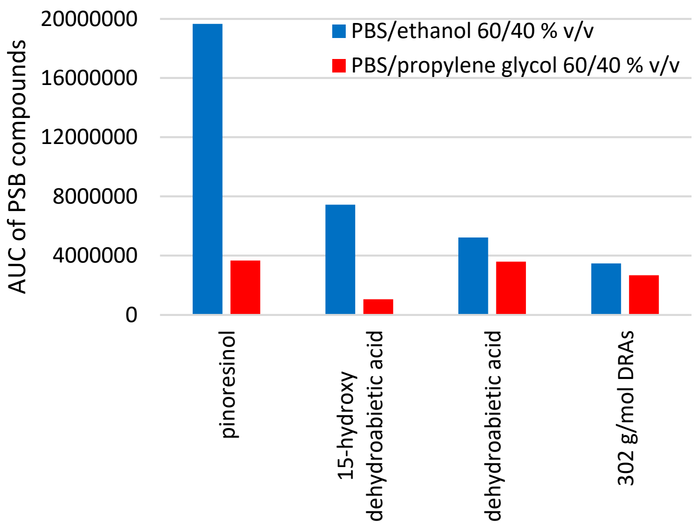

Figure S3. Comparison of the relative solubility of PSB compounds in different acceptor media. AUCs were obtained after dissolving an identical surplus amount of purified spruce balm (PSB) in either phosphate-buffered saline/ethanol at 60:40% *v*/*v* (blue bars) or phosphate-buffered saline/propylene glycol at 60:40% *v*/*v* (red bars).

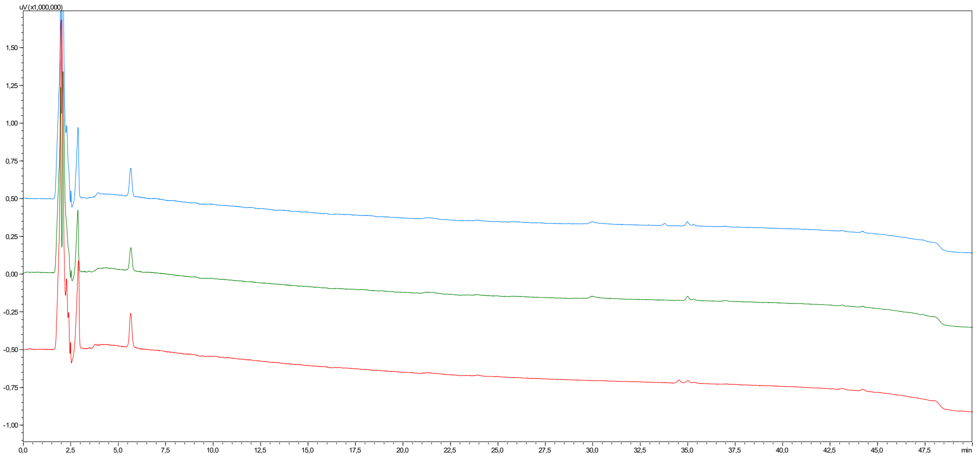

Figure S4. HPLC-DAD chromatograms of samples drawn from permeation experiments after 32 h using no ointment (red), pig fat without PSB (green), and wool wax/castor oil without PSB (blue) at 190 nm.

Table S1. Batch numbers (PSB), internal assignment (FP), date of harvest, and harvest location of analyzed PSB batches.

PSB batch	Date of harvest	Harvest location
**PSB 1** (FP6)	07/2016	Promau Kitzhütte, Hollenstein, Lower Austria
**PSB 2** (FP12)	07/2016	Gaflenz Heiligenstein, Upper Austria
**PSB 3** (FP13)	2015	Lungau, Salzburg
**PSB 4** (FP21)	2010-2016	Austria
**PSB 5** (FP23)	07/2021	Schellgaden, Tamsweg, Lungau, Salzburg
